# Type 2 NADH Dehydrogenase Is the Only Point of Entry for Electrons into the Streptococcus agalactiae Respiratory Chain and Is a Potential Drug Target

**DOI:** 10.1128/mBio.01034-18

**Published:** 2018-07-03

**Authors:** Andrea M. Lencina, Thierry Franza, Matthew J. Sullivan, Glen C. Ulett, Deepak S. Ipe, Philippe Gaudu, Robert B. Gennis, Lici A. Schurig-Briccio

**Affiliations:** a Department of Biochemistry, University of Illinois, Urbana, Illinois, USA; b Micalis Institute, INRA, AgroParisTech, Université Paris-Saclay, Jouy-en-Josas, France; c School of Medical Sciences and Menzies Health Institute Queensland, Griffith University, Gold Coast, Australia; Michigan State University; Nanyang Technological University

**Keywords:** bacterial pathogenesis, electron transport chain, NADH dehydrogenase, Streptococcus agalactiae, drug discovery

## Abstract

The opportunistic pathogen Streptococcus agalactiae is the major cause of meningitis and sepsis in a newborn’s first week, as well as a considerable cause of pneumonia, urinary tract infections, and sepsis in immunocompromised adults. This pathogen respires aerobically if heme and quinone are available in the environment, and a functional respiratory chain is required for full virulence. Remarkably, it is shown here that the entire respiratory chain of S. agalactiae consists of only two enzymes, a type 2 NADH dehydrogenase (NDH-2) and a cytochrome *bd* oxygen reductase. There are no respiratory dehydrogenases other than NDH-2 to feed electrons into the respiratory chain, and there is only one respiratory oxygen reductase to reduce oxygen to water. Although S. agalactiae grows well in *vitro* by fermentative metabolism, it is shown here that the absence of NDH-2 results in attenuated virulence, as observed by reduced colonization in heart and kidney in a mouse model of systemic infection. The lack of NDH-2 in mammalian mitochondria and its important role for virulence suggest this enzyme may be a potential drug target. For this reason, in this study, S. agalactiae NDH-2 was purified and biochemically characterized, and the isolated enzyme was used to screen for inhibitors from libraries of FDA-approved drugs. Zafirlukast was identified to successfully inhibit both NDH-2 activity and aerobic respiration in intact cells. This compound may be useful as a laboratory tool to inhibit respiration in S. agalactiae and, since it has few side effects, it might be considered a lead compound for therapeutics development.

## IMPORTANCE

S. agalactiae is part of the human intestinal microbiota and is present in the vagina of ~30% of healthy women. Although a commensal, it is also the leading cause of septicemia and meningitis in neonates and immunocompromised adults. This organism can aerobically respire, but only using external sources of heme and quinone, required to have a functional electron transport chain. Although bacteria usually have a branched respiratory chain with multiple dehydrogenases and terminal oxygen reductases, here we establish that S. agalactiae utilizes only one type 2 NADH dehydrogenase (NDH-2) and one cytochrome *bd* oxygen reductase to perform respiration. NADH-dependent respiration plays a critical role in the pathogen in maintaining NADH/NAD^+^ redox balance in the cell, optimizing ATP production, and tolerating oxygen. In summary, we demonstrate the essential role of NDH-2 in respiration and its contribution to S. agalactiae virulence and propose it as a potential drug target.

## INTRODUCTION

Streptococcus agalactiae (group B *Streptococcus* [GBS]) is a facultative, fermentative commensal bacterium normally living in the gut and urogenital tract of healthy individuals. It belongs to the family *Streptococcaceae*, many of which are opportunistic pathogens, and is able to transition to invasive niches, causing excessive inflammation, sepsis, and death (1). S. agalactiae is the major cause of meningitis and sepsis in a newborn’s first week of life in the United States, as well as a considerable cause of pneumonia and sepsis in immunocompromised adults (2). In neonates, S. agalactiae is transmitted by the mother via aspiration of fluids during birth. Although most transmission can be prevented by intravenous antibiotic administration during labor, allergies and emerging resistance to such antibiotics are an increasing concern (2). S. agalactiae is also associated with a large fraction of urinary tract infections in the elderly and nursing home residents, including kidney and bladder infections (3).

Despite its capacity for fermentative metabolism, S. agalactiae can perform aerobic respiration in the presence of external sources of heme and quinone. Within the same operon, the genome encodes a cytochrome *bd* oxygen reductase (cyt *bd* encoded by *cydAB*), a putative type 2 NADH dehydrogenase (NDH-2 encoded by *ndh*), and a 1,4-dihydroxy-2-naphthoate prenyltransferase enzyme (encoded by *menA*) (4–6). The *menA* gene is normally involved in the synthesis of demethylmenaquinone (DMK-10). However, genes other than *menA* that are required to synthesize menaquinone (MK) are not present in S. agalactiae, which is, therefore, not able to synthesize DMK-10 from chorismate (6). Disabling cyt *bd* (Δ*cydA*) results in decreased organ colonization and increased survival of neonatal rats compared to wild-type (WT) infection, indicating a link between respiration and virulence (4, 7).

NDH-2 is a homodimeric flavoprotein that catalyzes the oxidation of NADH with the concomitant reduction of quinone. It is a monotopic membrane enzyme that binds at the cytoplasmic surface of the bacterial membrane in order to have access to one of its substrates (quinone) but has no transmembrane domain (8–11). cyt *bd* is a transmembrane, heme-containing two-subunit enzyme (CydA and CydB) that catalyzes menaquinol:O_2_ oxidoreductase activity (12). The chemical reaction catalyzed by cyt *bd* results in the net electrogenic transfer of two protons from the cytoplasm to the extracellular space, contributing to the proton motive force (PMF) (12, 13). Both NDH-2 and cyt *bd* are absent in mammalian mitochondria, making them plausible drug targets (14). NDH-2, which plays an important role in pathogen survival and virulence, has been pursued as a possible drug target in Mycobacterium tuberculosis (15, 16), Toxoplasma gondii (17), and Plasmodium falciparum (18, 19).

**FIG 1:**
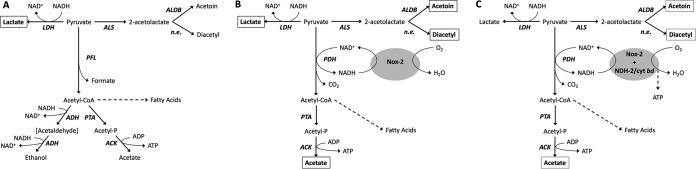
Pyruvate catabolism in S. agalactiae. One molecule of glucose will produce two of pyruvate via glycolysis, with net reduction of 2 NADH molecules. Fermentation of pyruvate, will allow NADH reoxidation for its recycling and use in a new round of glycolysis. (A) In the absence of oxygen, acetyl coenzyme A (acetyl-CoA) is made from pyruvate by pyruvate formate lyase (PFL), allowing synthesis of fatty acids. The main fermentation product is lactate, but significant amounts of ethanol, acetate, acetoin, and diacetyl are also found (21). Under aerobic conditions, production of acetoin, diacetyl, and acetate is prevalent over that of ethanol. (B) In the presence of oxygen without addition of external heme or quinone sources (respiration nonpermissive condition), lactate is still the main fermentation product, which leads to a significant decrease in pH. (C) Upon addition of external heme and quinone, in the presence of oxygen (respiration permissive condition), the respiratory chain (NDH-2/cyt *bd*) becomes functional. Metabolism is shifted toward production of acetate, acetoin, and diacetyl, reducing the amount of pyruvate available to be converted into lactate. This results in less acidification of the medium in the stationary phase. Higher growth yield is achieved as a result of enhanced ATP formation via the Pta-Ack pathway and possibly oxidative phosphorylation by the electron transport chain. Boxes indicate the main products for each condition. ACK, acetate kinase; ALS, acetolactate synthase; ALDB, 2-acetolactate decarboxylase; LDH, lactate dehydrogenase; PDH, pyruvate dehydrogenase; PTA, phosphate transacetylase; n.e., nonenzymatic reaction. (Adapted with data from references 21 to 23 and 66.)

To understand the significance of NDH-2 in S. agalactiae survival and virulence and the consequences of its deficiency, it is important to consider the main metabolic strategies used by this pathogen (Fig. 1). Glycolysis yields 2 eq each of pyruvate and NADH. Growth requires not only ATP production but also a way to recycle NADH to NAD^+^ to allow glycolysis to proceed. Figure 1 shows alternative pathways for pyruvate catabolism in S. agalactiae that contribute to different degrees, depending on growth conditions. Note that this organism does not have the enzymes required for the tricarboxylic acid (TCA) cycle (20).

Under anaerobic growth conditions, S. agalactiae must grow strictly by fermentation (21–23). Under growth conditions in which sugar metabolism is rapid and there is constant NADH production, NAD^+^ is regenerated by converting pyruvate to lactate via lactate dehydrogenase (homolactic fermentation) (Fig. 1A). Some growth conditions, however, result in a shift toward mixed-acid fermentation, in which pyruvate can be catabolized by pyruvate dehydrogenase, pyruvate formate lyase, and/or acetolactate synthase, yielding a variety of end products, including formate/CO_2_, acetate, and acetoin (Fig. 1B). The presence of oxygen has several consequences. First, pyruvate formate lyase is particularly susceptible to inactivation by oxygen. Second, if both heme and quinone can be acquired from the surroundings, S. agalactiae utilizes its respiratory chain to regenerate NAD^+^, generate a PMF, enhance ATP production via acetate kinase (Ack) and possibly oxidative phosphorylation, and reduce the concentration of O_2_ (Fig. 1C). Finally, S. agalactiae also has a water-forming NADH oxidase (Nox-2) (23), a soluble enzyme that reduces oxygen to water and regenerates NAD^+^ but does not contribute to the energy needs of the cell. In the absence of heme and quinone, S. agalactiae relies on Nox-2 for recycling NAD^+^ to feed glycolysis, to synthesize fatty acids, and for reduction of oxygen to water.

In this study, it is demonstrated that NDH-2 is an essential element for aerobic respiration and that disabling this enzyme reduces virulence in a mouse model of systemic infection. In addition, the S. agalactiae NDH-2 is purified, biochemically characterized, and proven a potential drug target for GBS.

## RESULTS

### S. agalactiae *ndh* encodes a highly active NDH-2.

**FIG 2:**
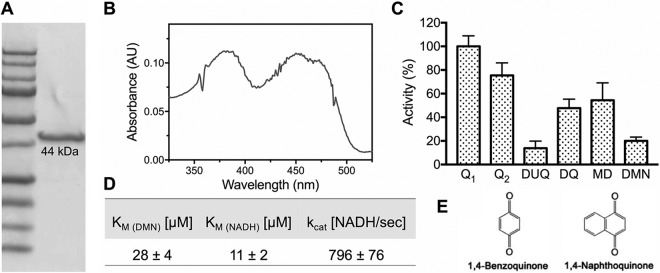
The isolated enzyme from S. agalactiae is a highly active NDH-2. (A) SDS-PAGE of ~5 µg of isolated protein. (B) UV-Vis spectrum of the flavin. (C) Enzyme activity was measured with different soluble quinone analogues. Data are expressed as average ± standard deviation (SD) from three independent experiments. Q_1_, ubiquinone-1; Q_2_, ubiquinone-2; DUQ, decylubiquinone; DQ, duroquinone; MD, menadione; DMN, 2,3-dimethyl-1,4-naphthoquinone. One hundred percent activity corresponds to 4,190 NADH s^−1^ observed in the presence of Q_1_. (D) Turnover number (*k*_cat_) and affinity (*K*_*m*_) for the enyzme’s substrates were determined. Data are expressed as average ± SD from three independent experiments. (E) Core structures for benzoquinones (Q_1_, Q_2_, DUQ, and DQ) and naphthoquinones (MD and DMN).

Generally, the low amino acid sequence identity among NDH-2s makes it difficult to distinguish them from other small, soluble flavoenzymes (24–26). The S. agalactiae *ndh* gene encodes a putative NDH-2 enzyme, showing relatively high identity to Staphylococcus aureus NdhC (42%) (27) and Caldalkalibacillus thermarum NDH-2 (41%) (see Fig. S1 in the supplemental material) (10, 11). The S. agalactiae *ndh* gene with an N-terminal 8×His tag was cloned and heterologously expressed in E. coli c43. S. agalactiae NDH-2 was purified, and SDS-PAGE analysis shows a single band at the predicted molecular weight of 44 kDa (Fig. 2A). The UV-visible (UV-Vis) spectrum of the air-oxidized protein shows characteristic peaks for flavin adenine dinucleotide (FAD) around 375 and 450 nm, with a shoulder around 470 nm indicating the cofactor is in an apolar environment (28) (Fig. 2B). The FAD cofactor remains in solution after precipitation of the protein with 5% trichloroacetic acid, demonstrating the flavin is noncovalently bound (29). Quantitation of the extracted flavin was calculated to be 0.25 mol FAD/mol protein. Substoichiometric flavin content is often observed with heterologously expressed flavoproteins (27, 30). Since the protein produced in E. coli shows substoichiometric amounts of FAD, assays were performed in the presence of 20 µM FAD. Upon addition of the flavin, a 10-fold increase in activity was observed. All results reported were performed with 20 µM FAD in the assay buffer.

10.1128/mBio.01034-18.1 FIG S1  S. agalactiae amino acid sequence shows high identity to C. thermarum and S. aureus NDH-2s. Identical residues among the three sequences are shaded in red. The figure was made using ESPript (67). Download FIG S1, TIF file, 1.4 MB. Copyright © 2018 Lencina et al. 2018 Lencina et al. This content is distributed under the terms of the Creative Commons Attribution 4.0 International license.

Although lacking any transmembrane-spanning elements, NDH-2 is a membrane-bound protein that, upon isolation, requires the presence of detergent to remain in solution. Optimal enzyme activity and stability also required 150 to 300 mM NaCl and pH 7. NADH:quinone oxidoreductase activity was tested in the presence of NADH and different soluble quinone analogues (Fig. 2C). The S. agalactiae endogenous electron acceptor for NDH-2 is DMK-10 (6), a naphthoquinone, but the pure enzyme is also active with ubiquinones (Fig. 2C and E). With 2,3-dimethyl-1,4-naphthoquinone (DMN), the soluble menaquinone analogue most similar to the natural electron acceptor in S. agalactiae membranes, the turnover number is about 800 NADH/s^−1^ and the *K*_*m*_ values for DMN and NADH are 11 µM and 28 µM, respectively. The isolated S. agalactiae NDH-2 does not react directly with O_2_ in the presence of NADH, unlike the NDH-2 from Corynebacterium glutamicum (31).

### NDH-2 is the only entry point for electrons into the respiratory chain of S. agalactiae*.*

An *ndh* deletion (Δ*ndh*) mutant was constructed to characterize the role of NDH-2 in the S. agalactiae respiratory metabolism. Since both aerobic respiration and Nox-2 are involved in oxygen reduction to water and regeneration of NAD^+^ (23) (Fig. 1), a Δ*ndh* Δ*nox2* double mutant strain was also constructed to characterize the effect on metabolism and oxygen tolerance. As a control, a previously studied *nox2* single-knockout strain (23) was also examined.

**FIG 3:**
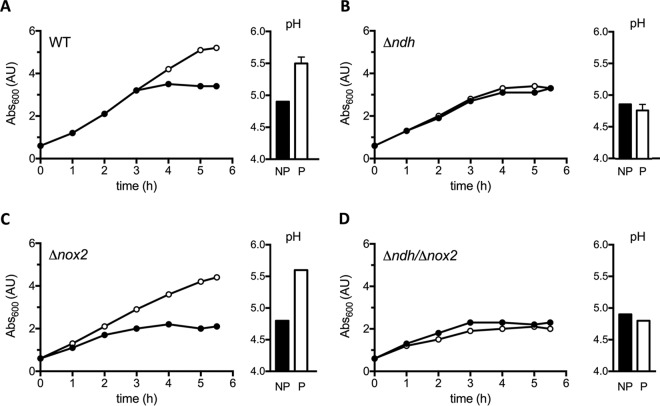
The NDH-2-deficient strain shows a nonrespiratory phenotype. Growth curves and pH of the media at 8 h of culture are shown for the WT (A) and the Δ*ndh* (B), Δ*nox2* (C), and Δ*ndh* Δ*nox2* (D) mutant strains. Cells were grown under respiration permissive (P [white circles and bars]) and nonpermissive (NP [black circles and bars]) conditions. Growth curves are representative of at least 3 independent experiments. pH data are plotted as the average ± SD from three independent experiments.

The WT, Δ*ndh*, Δ*nox2*, and Δ*ndh* Δ*nox2* strains were grown aerobically in the presence of externally added heme and quinone (referred to as the respiration permissive condition) and in the absence of heme and quinone (referred to as the respiration nonpermissive condition) (Fig. 3). When the WT or Δ*nox2* mutant strain is grown aerobically in the presence of external heme and quinone, enhanced ATP production results in an improved growth yield (Fig. 3A and C), and there is a metabolic shift favoring mixed-acid fermentation, reduced lactate formation, and hence, a smaller pH drop in the medium (4, 32). In contrast, the Δ*ndh* strain does not show any difference in growth rate or pH drop (Fig. 3B), likely due to an increase in consumption of glucose that results in a higher production of lactate (which has a lower pK_a_) observed under nonpermissive conditions. This is to overcome the lower energetic efficiency derived from homolactic fermentation. The growth behavior and pH of the Δ*ndh* strain are restored to that of the WT strain if the deletion is complemented by a plasmid carrying the *ndh* gene under the control of its own promoter (see Fig. S2 in the supplemental material), indicating the observed effect is in fact due to NDH-2 activity. Similar results are observed for the Δ*ndh* Δ*nox2* strain (Fig. 3D).

10.1128/mBio.01034-18.2 FIG S2  The Δ*ndh* growth phenotype is complemented by a plasmid-carried copy of *ndh*. (A and B) Final growth (A) and pH (B) at 8 h of culture under respiration permissive (white bars) or nonpermissive (black bars) conditions for WT and Δ*ndh* strains carrying the empty vector (p) or *ndh*-expressing vector (p-*ndh*). Data are plotted as the average ± SD from three independent experiments. *, *P* = 0.0001 via two-way ANOVA with Dunnett’s posttest compared to wild-type nonpermissive. Download FIG S2, TIF file, 0.2 MB. Copyright © 2018 Lencina et al. 2018 Lencina et al. This content is distributed under the terms of the Creative Commons Attribution 4.0 International license.

**FIG 4:**
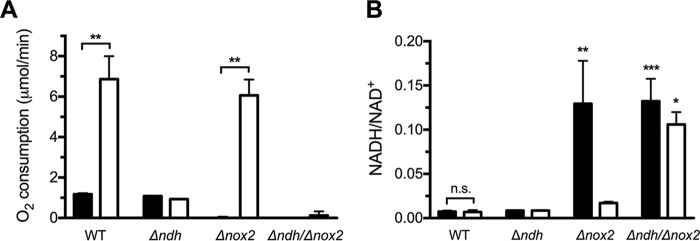
NDH-2 is important for respiration and for maintenance of NADH/NAD ^**+**^ redox balance. Cells were grown under respiration permissive (white bars) or nonpermissive (black bars) conditions and harvested in early stationary phase. (A) Oxygen consumption assays were normalized to an OD_600_ of 0.3 and started by the addition of 0.2% glucose. (B) The NADH/NAD^+^ ratio was determined for the WT and isogenic mutant strains. Data are plotted as the average ± SD from three independent experiments. (A) **, *P* < 0.005 via two-way analysis of variance (ANOVA) with Tukey’s posttest for the indicated comparisons. No significant difference was observed between nonpermissive and permissive conditions for the Δ*ndh* or Δ*ndh* Δ*nox2* mutants. (B) n.s., not significant, *, *P* < 0.05, **, *P* < 0.005, and ***, *P* < 0.001, via two-way ANOVA with Dunnett’s posttest compared to wild-type under nonpermissive conditions.

The effect of the deletions on oxygen utilization was tested for cells grown under either respiration permissive or nonpermissive conditions (Fig. 4A). Both respiration (NDH-2/cyt *bd*) and Nox-2 activity result in oxygen utilization. In the Δ*ndh* Δ*nox2* double deletion strain, no oxygen utilization is observed under any circumstances, suggesting that the elimination of NDH-2 results in the complete absence of respiration.

When grown in the absence of heme and quinone, the WT and Δ*ndh* strains utilize oxygen at the same rate, since Nox-2 is the only enzyme contributing to the measured depletion of O_2_. In the presence of heme and quinone, the WT strain has substantially more activity than the Δ*ndh* strain, since the WT strain is able to respire, while the Δ*ndh* strain cannot. All of the observed oxygen utilization of the Δ*nox2* strain, grown with heme and quinone, is due to NDH-2/cyt *bd* respiration. Under the conditions of these experiments and comparing the Δ*ndh* and Δ*nox2* strains, the capacity for O_2_ utilization by the respiratory chain can be estimated to be about 6-fold higher than that for Nox-2 (Fig. 4A).

Lastly, the importance of aerobic respiration and Nox-2 in NADH reoxidation was evaluated in cells grown under respiration permissive or nonpermissive conditions (Fig. 4B). When grown under permissive conditions, the NADH/NAD^+^ ratio did not show significant differences in the Δ*ndh* and Δ*nox2* strains. However, both the Δ*ndh* Δ*nox2* strain and the Δ*nox2* strain, when grown under nonpermissive conditions, show a drastic increase in NADH and the NADH/NAD^+^ ratio compared to the wild type. This suggests that NDH-2 (respiration) and Nox-2 are the major routes for NAD^+^ recycling, and these observations support the previously proposed role for respiration in fatty acid biosynthesis (23).

### NDH-2 is important for S. agalactiae virulence.

**FIG 5:**
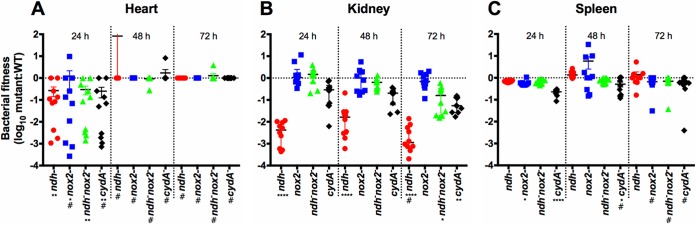
The Δ*ndh*, Δ*nox2*, and Δ*cydA* mutant strains are attenuated for virulence in heart, kidney, and spleen. Mice were infected with 2 × 10^6^ CFU of S. agalactiae, and bacteria were counted at 24, 48, and 72 h postinfection in heart (A), kidney (B), and spleen (C). Bacterial fitness of mutants is shown as ratio of the log_10_ number (CFU/gram) of mutant to WT bacteria. *, *P* < 0.05, **, *P* < 0.01, and ****, *P* < 0.0001 (mutant/WT ratio), by Kruskal-Wallis test followed by Dunn’s multiple comparisons. # denotes a median bacterial load (CFU per milliliter) of zero for the group.

**FIG 6:**
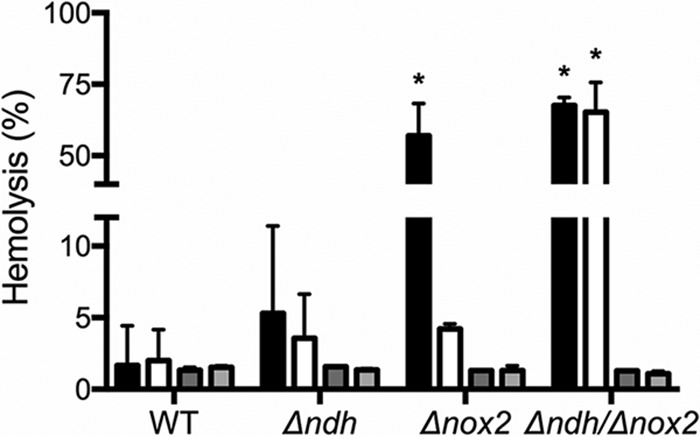
Hemolytic activity correlates with NADH/NAD^**+**^ ratios. Cells were grown under respiration permissive (white bars) or nonpermissive (black bars) conditions until the early stationary phase, then washed and incubated with RBCs for 1 h. Dark and light gray bars represent respiration nonpermissive and permissive growth, respectively, with 0.1% Tween 80 added to the media. Data are plotted as the average ± standard error of the mean (SEM) from three independent experiments. *, *P* < 0.0001 via two-way ANOVA with Dunnet’s posttest compared to the wild type under nonpermissive conditions.

Due to the essential role of NDH-2 in S. agalatiae respiration, we investigated its contribution to the development of invasive disease. The bacterial burdens or fitness of the S. agalactiae WT and mutant strains in different organs were determined after 24, 48, and 72 h postinfection (Fig. 5). Infection assays using the S. agalactiae Δndh strain and Δ*ndh* Δ*nox2* double mutant strain revealed significant attenuation in heart and kidney colonization compared to WT (Fig. 5A and B). The Δ*nox2* strain is significantly attenuated in heart and spleen compared to the WT (Fig. 5A and C), as reported in previous studies (23). Unexpectedly, no pronounced phenotype is seen in any organ with the Δ*ndh* Δ*nox2* double mutant strain, although this double mutant does not tolerate oxygen well. In an effort to explain this, we investigated the hemolytic activity of these knockout strains (Fig. 6). The cell surface-associated β-hemolysin/cytolysin of S. agalactiae is a nonimmunogenic, oxygen-stable, pore-forming cytolysin and a red polyenic pigment (33, 34). This major virulence factor is encoded by the *cylE* gene in the *cyl* locus, a unique 12-gene operon involved in fatty acid biosynthesis (35) that is expressed by most GBS strains. This virulence factor has proapoptotic, proinflammatory, and cytotoxic effects and is necessary for full S. agalactiae virulence in multiple *in vivo* systems (36). Furthermore, the hemolytic pigment has been shown to protect cells against a panel of stresses (33, 37). Remarkably, increased hemolysis was observed for the double mutant compared to the Δ*ndh* strain (Fig. 6), which correlates with the dramatic increase seen for the NADH/NAD^+^ ratio (Fig. 4B). Thus, this could contribute to the rescue effect observed during infection. Consistent with this interpretation, hemolysis is restored to WT levels upon addition of 0.1% Tween 80 to the cell cultures, effectively providing fatty acids to the cells (Fig. 6).

Among the mutants tested, the strain lacking cyt *bd* (Δ*cydA*) has been previously shown to result in changes in organ colonization in mice (7). The *cydA* knockout strain, used here as a control for S. agalactiae attenuation in the model of systemic infection, is significantly attenuated in heart, kidney, and spleen (Fig. 5), as well as liver (see Fig. S3 in the supplemental material), and its attenuation approaches statistical significance in blood (*P* = 0.08) (Fig. S3), findings consistent with what has been previously observed (7). None of the mutants is significantly attenuated in blood, liver, or brain colonization (Fig. S3A to C). These data support the conclusion that respiration is important for virulence in S. agalactiae (4, 7) and show, furthermore, that NDH-2 contributes to virulence.

10.1128/mBio.01034-18.3 FIG S3  The Δ*ndh*, Δ*nox2*, and Δ*cydA* mutant strains are not attenuated for virulence in blood, liver, or brain. Mice were infected with 2 × 10^6^ CFU of S. agalactiae, and bacteria were enumerated at 24, 48, and 72 h postinfection in blood (A), liver (B), and brain (C). Bacterial fitness of mutants is shown as a ratio of the log_10_ number (CFU/gram) of mutant to WT bacteria. *, *P* < 0.05 for the mutant/WT ratio by Kruskal-Wallis test followed by Dunn’s multiple comparisons. # denotes a median bacterial load (CFU/milliliter) of zero for the group. Download FIG S3, TIF file, 0.3 MB. Copyright © 2018 Lencina et al. 2018 Lencina et al. This content is distributed under the terms of the Creative Commons Attribution 4.0 International license.

### Inhibitors of S. agalactiae NDH-2 are potential leads against GBS.

Since this work demonstrates that NDH-2 is essential for respiration in S. agalactiae as well as important for virulence, inhibitors of the isolated enzyme were obtained and then tested as inhibitors of respiration in intact S. agalactiae cells. Enzymatic activity was monitored colorimetrically in the presence of NADH and menadione (MD), a commercially available soluble analogue of menaquinone. Two libraries of FDA-approved drugs were screened (about 2,000 compounds) using 25 µM each compound to test for inhibitors of the pure enzyme. Nine compounds inhibited at least 80% of enzymatic activity: alexidine, candesartan, caspofungin, closantel, hexachlorophene, isoquercetin, pramoxine, triclabendazole, and zafirlukast. Except for hexachlorophene, a relatively toxic disinfectant, all other compounds were tested against whole cells growing under respiration permissive conditions, and five of these showed an inhibitory effect on cell growth (Table 1).

**TABLE 1:** FDA-approved compounds that inhibit NDH-2 activity and S. agalactiae growth *in vitro*^a^

Compound	IC_50_ (µM)	GI_50_ (µM)	Common use
Alexidine	16.79 ± 1.26	2.70 ± 0.51	Antiseptic
Caspofungin	15.23 ± 2.33	41.03 ± 2.62	Antifungal
Closantel	5.34 ± 0.65	0.25 ± 0.04	Antihelmintic (veterinary)
Triclabendazole	0.14 ± 0.02	15.30 ± 1.92	Antihelmintic
Zafirlukast	0.83 ± 0.07	6.07 ± 0.10	Asthma treatment

a Cells were grown under respiration permissive conditions.

Although candesartan, isoquercetin, and pramoxine are able to inhibit the purified enzyme *in vitro*, they show no effect on cell growth. On the other hand, alexidine and closantel inhibit cell growth at concentrations much lower than those required for NDH-2 inhibition, suggesting other targets might be responsible for their effect in whole cells. This is not surprising since both compounds are known to disturb membrane integrity, producing permeabilization (38) and strong uncoupling (39). Finally, caspofungin, triclabendazole, and zafirlukast not only show NDH-2 inhibition in the low micromolar and even nanomolar range but are also able to inhibit cell growth at low micromolar concentrations (Table 1).

**FIG 7:**
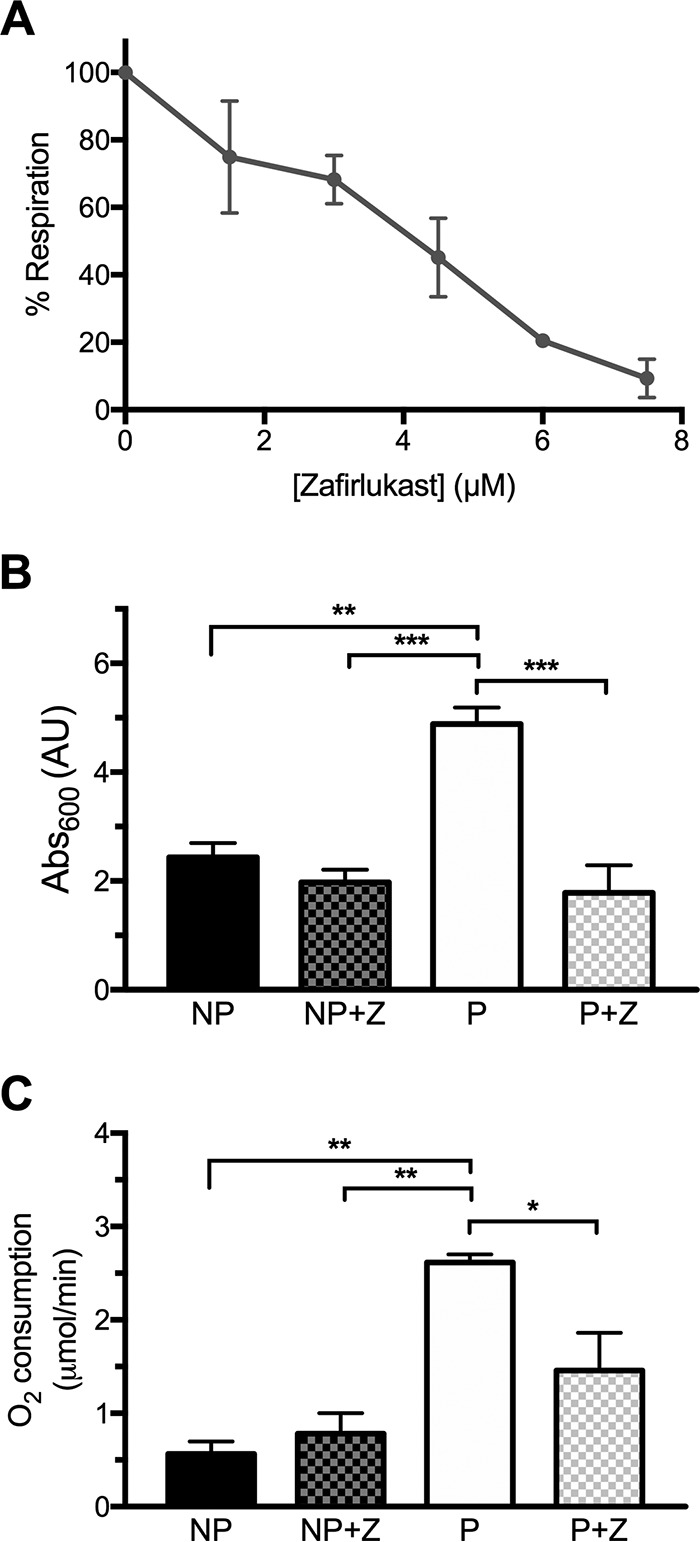
Zafirlukast inhibits S. agalactiae respiration. Cells were grown until the early stationary phase (8 h), and the percentage of respiration was calculated after addition of different concentrations of zafirlukast (A). The percentage of respiration corresponds to the difference in oxygen consumption of cells grown under respiration permissive (P) minus nonpermissive (NP) conditions. (B and C) Overnight growth (B) and oxygen consumption (C) of the Δ*nox2* mutant strain in the absence or presence of 7.5 µM zafirlukast (Z). Data are plotted as the average ± SD from three independent experiments. In panels B and C, *, *P* < 0.05, **, *P* < 0.005, and ***, *P* < 0.001, via two-way ANOVA with Tukey’s posttest for the indicated comparisons. No significant difference was observed between conditions NP, NP+Z, and P+Z.

Additional assays followed oxygen consumption of WT cells (grown with heme and quinone) in the presence of different concentrations of caspofungin, triclabendazole, or zafirlukast. From these compounds, only zafirlukast shows a dose-dependent linear decline in respiration, with 90% inhibition at 7.5 µM (Fig. 7A). In addition, the Δ*nox2* strain was grown overnight with and without subinhibitory concentrations of zafirlukast (~50% growth inhibitory concentration [GI_50_]), and both growth and respiration were examined after overnight incubation. The Δ*nox2* strain grown in the presence of zafirlukast does not exhibit enhanced growth under respiration permissive conditions compared to nonpermissive conditions. This indicates that the bacterial growth supported by the activity of the electron transport chain is substantially reduced in the presence of zafirlukast (Fig. 7B). Not all of the NDH-2 activity was eliminated since the cells were grown with a concentration of zafirlukast that does not completely inhibit the enzyme. This is shown in Fig. 7C where the oxygen utilization, which must be due to respiration in the Δ*nox2* strain, is substantially lower than when the cells are grown in the absence of zafirlukast, but not as low as when cells are grown in the absence of heme and quinone. In sum, the data show that zafirlukast inhibits the activity of the isolated S. agalactiae NDH-2 as well as the activity of the enzyme in intact cells.

## DISCUSSION

S. agalactiae can cause invasive disease in newborns leading to meningitis and septicemia and is also associated with urinary tract infections in the elderly population. It is a highly adapted organism capable of metabolizing a wide variety of substrates; however, it is an obligate commensal, auxotrophic for several amino acids and vitamins (1, 20). Although it used to be considered an aerotolerant organism, S. agalactiae was found over 10 years ago to perform aerobic respiration in the presence of external sources of heme and quinone (4). Aerobic respiration consists of the transport of electrons through a series of redox components, from a metabolic reductant (e.g., NADH) and terminating in the reduction of oxygen to water by a terminal oxygen reductase. Importantly, respiration is linked to the generation of PMF and ATP synthesis via Ack and possibly oxidative phosphorylation (Fig. 1C).

### S. agalactiae has a two-enzyme respiratory chain.

S. agalactiae has a single oxygen reductase, cyt *bd*. The first conclusion of the present work is that there is only one enzyme, NDH-2, that provides electrons to the respiratory chain of this organism. Both NDH-2 and cyt *bd* are encoded by genes (*ndh* and *cydAB*, respectively) within the same operon. Although single-subunit respiratory flavoenzymes are often misannotated (40), an exhaustive search of the genome of S. agalactiae shows no other genes for any additional respiratory enzymes, like glycerol-3-P dehydrogenase, pyruvate oxidase, or membrane-bound succinate, lactate, or malate dehydrogenases. A *glp* operon for glycerol metabolism is present; however, glycerol phosphate dehydrogenase (*glpD*) is replaced by a glycerol phosphate oxidase (*glpO*), as in other organisms that do not synthesize heme. The latter enzyme is soluble and reduces oxygen to hydrogen peroxide instead of reducing quinone (41). Also, whereas some bacterial pathogens (e.g., S. aureus) have two different NDH-2 enzymes (27), S. agalactiae has only one, and there is no other type of respiratory NADH dehydrogenase, such as complex I (NDH-1) or the sodium pumping NDH (Nqr) (42). Thus, unlike most other bacteria, the electron transport chain of S. agalactiae is not branched at either end and consists of just two proteins plus DMK-10 (6).

### The *ndh* gene encodes an authentic NDH-2.

The S. agalactiae *ndh* gene was cloned and expressed in E. coli, and the purified recombinant protein was shown to be an NADH:quinone oxidoreductase. The isolated, recombinant S. agalactiae NDH-2, which has an amino acid sequence identity of 42% with S. aureus NdhC, has a high *k*_cat_ (~800 NADH s^−1^) that is similar to that of S. aureus NdhC (~1,500 NADH s^−1^) (27). These *k*_cat_ values are substantially higher than those reported for NDH-2s from other organisms, including the NDH-2s from E. coli, with a *k*_cat_ of ~18 NADH s^−1^ (43), M. tuberculosis, with a *k*_cat_ of ~10 NADH s^−1^ (44), Bacillus pseudofirmus, with a *k*_cat_ of ~39 NADH s^−1^ (45), Bacillus subtilis, with a *k*_cat_ of ~20 NADH s^−1^ (46), and C. glutamicum, with a *k*_cat_ of ~213 NADH s^−1^ (31). The high activities obtained for the enzymes from S. agalactiae and S. aureus are most likely due to the presence of phospholipids and FAD in the reaction buffer of our assays. The S. agalactiae NDH-2 has a lower *K*_*m*_ for NADH (11 µM) than those of other previously characterized NDH-2s from bacteria. For example, the apparent *K*_*m*_ value for NADH of the NDH-2 from E. coli is 34 µM (43), that from B. subtilis is 60 µM (46), that from B. pseudofirmus is 114 µM (45), and those from S. aureus are 35 µM (NdhC) and 154 µM (NdhF) (27).

### Multiple advantages of utilizing aerobic respiration.

S. agalactiae grows well by utilizing a variety of fermentation strategies (Fig. 1), but the operon encoding NDH-2 and cyt *bd* is expressed constitutively so the cells can very rapidly switch to respiratory growth (4) in the presence of O_2_ after taking up quinone and heme from the environment (6, 47). Certainly, a major advantage of respiratory growth is a higher bioenergetics capacity via Ack and possibly oxidative phosphorylation. In addition to enhanced growth efficiency, respiration also serves for (i) reduction of the intracellular and local concentrations of O_2_ and to combat oxygen toxicity, (ii) generation of NAD^+^ necessary for glycolysis and fatty acid synthesis (23), (iii) generation of the PMF required for powering transporters and essential efflux pumps like PefAB (13, 48), (iv) contribution to resistance against environmental acid stress (5, 49), and (v) production of virulence factors (e.g., respiration is required for nuclease production) (50).

### Respiration is linked to virulence and organ colonization.

The different stimuli found in the growth environment, including the carbon source(s), concentration of O_2_, and the availability of external heme and quinone, determine the metabolic pathways utilized to optimize the growth and survival of S. agalactiae. The growth conditions vary significantly when comparing *in vitro* batch growth to *in vivo* growth in a host animal or when comparing the *in vivo* growth in different organs. The combination of metabolic pathways utilized by S. agalactiae must simultaneously generate ATP, maintain the PMF, reduce levels of O_2_ if it is present, and maintain the recycling of NADH through NAD^+^. Since the metabolic strategies used by S. agalactiae can be very different in each organ in an infected animal, it is reasonable that mutations that incapacitate respiration will have different effects on organ colonization, depending on the organ. Based on studies with a cyt *bd* oxidase deletion mutant, S. agalactiae respiration has been previously linked to virulence and colonization of blood-rich organs (4, 7). It is shown in the present work that the deletion of the gene encoding NDH-2 eliminates *in vitro* respiration and also results in attenuation of organ colonization in a mouse model for systemic infection. The strongest attenuation from the *ndh* deletion is in heart and kidney. The apparent significance of respiration for kidney infection may be useful considering the importance of S. agalactiae in urinary tract infections in the elderly (3). Moreover, S. agalactiae is able to synthesize its own DMK-10, starting from 1,4-DHNA (dihydroxy-2-naphthoic acid) or short-chain quinones, such as MK-4 (6), which can be found in high concentrations in the kidney (51). An unexpected finding is that the Δ*ndh* Δ*nox2* double mutant strain is much less attenuated in kidney colonization than the highly attenuated Δ*ndh* strain (Fig. 5B). This may be related to the increased hemolysis observed for the double mutant strain compared to the Δ*ndh* strain (Fig. 6). The Δ*ndh* Δ*nox2* double mutant strain is highly impaired in NADH oxidation to NAD^+^ (Fig. 4B), and the deficiency of NAD^+^ likely results in impaired fatty acid biosynthesis (23). The increased hemolysis observed in the double-knockout strain (Fig. 6) may be the response of S. agalactiae to obtain fatty acids from surrounding host cells (52).

It is noteworthy that the Δ*ndh* strain is much more impaired in colonization in the kidney than is the Δ*cydA* strain, since both of these mutations disrupt respiration. One possible explanation is that the presence of a heme-containing enzyme in the membrane (cyt *bd*) in the Δ*ndh* strain makes the bacterial cells more susceptible to the strong oxidative stress displayed by the kidney during sepsis (53). Alternatively, higher levels of reduced menaquinone in the Δ*cydA* strain could lead to production of small amounts of intracellular reactive oxygen species (ROS), stimulating a response against oxidative stress through PerR (54, 55), enhancing survival of the strain in the kidney. The precise molecular explanation for the organ-specific effects of the mutants on colonization will require further studies.

### Inhibitors of S. agalactiae NDH-2.

Screening for inhibitors against the purified S. agalactiae NDH-2 was done in the presence of lipids to mimic the hydrophobic environment of the enzyme *in situ*. Although several compounds from libraries of FDA-approved drugs were found to inhibit the enzyme *in vitro*, only zafirlukast also inhibits respiration in intact cells in a way that suggests NDH-2 is the likely target *in vivo*. However, it is possible that this compound has other targets in S. agalactiae. Zafirlukast is a cysteinyl leukotriene receptor antagonist, currently used for the prophylaxis and chronic treatment of asthma (56). It is a commercially available generic drug and safe for daily use (57), and it has also been described to have bactericidal activity against M. tuberculosis by inhibiting complex formation between Lsr2 (a nucleoid-associated protein) and DNA (58). Zafirlukast also has been demonstrated to have antibacterial and antibiofilm activity against the nonrespiratory oral pathogens Porphyromonas gingivalis and Streptococcus mutans (59).

It is worth mentioning that despite its high similarity to S. aureus NdhC, S. agalactiae NDH-2 is not inhibited by trifluoperazine or thioridazine, phenothiazines that both show a strong inhibitory effect on S. aureus NdhC (27). These data demonstrate that the NDH-2 enzymes from different organisms, though apparently very similar, can have different inhibition profiles with drugs. These small enzymes, with binding sites for both NADH and quinol, appear to be susceptible to inhibitors that do not bind at the substrate binding sites but, rather, to allosteric sites (44). Although the substrate binding sites may be conserved among different NDH-2s, inhibitor-binding regions may differ greatly. In any event, zafirlukast may be useful as a laboratory tool to inhibit respiration in S. agalactiae and, since it has few side effects (60), might be considered a lead compound for therapeutics.

## MATERIALS AND METHODS

### Sequence analysis.

Gene sequences encoding S. agalactiae (gbs1788), S. aureus (SAB0708) and C. thermarum (GenBank accession no. ZP_08531709.1) NDH-2s were retrieved from the National Center for Biotechnology Information (NCBI) database. Amino acid sequence alignments were performed using ClustalW (61).

### Bacterial strains and growth conditions.

Strains and plasmids used in this work are listed in Table 2. S. agalactiae NEM316, a capsular serotype III strain with a fully sequenced genome, was used as the WT (20, 62). For growth in liquid media, cells were initially grown overnight at 37°C in M17 broth supplemented with 0.2% glucose and 80 µM menaquinone (MK-4), under static microaerobic conditions. These overnight cultures were used to inoculate tubes with M17 broth (1/10 vol/vol ratio) supplemented with 5 µM riboflavin, to an initial optical density at 600 nm (OD_600_) of ≈0.05. Culture tubes were incubated at 37°C—first statically until they reached an OD_600_ of ≈0.5 and then transferred to high-aeration conditions (200 rpm). To support respiration, 50 µg ml^−1^ of hemoglobin (in 0.9% NaCl aqueous solution) and 30 µM MK-4 were added to the cultures before transfer.

**TABLE 2:** Bacterial strains and plasmids used in this study

Strain or plasmid	Main characteristic(s)^a^	Reference or source
Strains		
S. agalactiae		
NEM316	Serotype III isolated from neonatal blood culture	20
Δ*ndh* mutant	NEM316 with *ndh* gene deleted	This work
Δ*nox2* mutant	NEM316 *nox2* Ω*aphA-3* Δ*nox2* Km^r^	23
Δ*ndh* Δ*nox2* mutant	Δ*nox2* mutant with *ndh* gene deleted, Km^r^	This work
NEMJ01	NEM316 *cydA*::*aphA-3* Km^r^	4
E. coli		
TG1	Host strain for sequencing	Lucigen
TOP10	Host strain for molecular cloning	Invitrogen
c43(DE3)	Host strain for protein overproduction	Avidis, France
Plasmids		
pBR322-pGhost8	Thermosensitive vector, Tet^r^	63
pTCV-lac	Shuttle vector, Ery^r^ Km^r^	64
pTCV-*ndh*^C^	*ndh* complementation plasmid	This work
pET-22b	Cloning vector, Amp^r^	Novagen

a Amp^r^, Km^r^, Tet^r^, and Ery^r^ indicate resistance to ampicillin, kanamycin, tetracycline, and erythromycin, respectively.

### Construction of *ndh*-deficient mutant and complementation plasmid.

Primers used for constructions are listed in Table 3. Chromosomal DNA from S. galactiae strain NEM316 was used as a template to amplify by PCR a DNA fragment upstream of the gene *gbs1788* (*ndh*) with primer pair gbs1788F and gbs1788intR. A second fragment downstream of *gbs1788* was obtained with primer pair gbs1788intF and gbs1788R. Both fragments were fused by PCR with gbs1788F and gbs1788R to give the fragment Δ*ndh*, which was further cloned into EcoRI and BamHI sites of the thermosensitive plasmid pBR322-pGhost8 (63) to give the plasmid pBR322-pGhost8-Δ*ndh*. The resulting plasmid was established in E. coli strain TG1 for sequencing and then transferred into the S. agalactiae NEM316 WT and Δ*nox2* mutant strains (23). S. agalactiae transformants were selected at 30°C on brain heart infusion (BHI) plates supplemented with 3 µg/ml tetracycline. The plasmid was integrated in the *ndh* locus when cells were grown at 37°C (first recombination event), followed by growth at 30°C for excision (second recombination event). Gene deletion was confirmed by PCR with primer pair gbs1788extF and gbs1788extR, designed outside the recombination region. In the Δ*ndh* strain, 373 of 402 amino acid residues of NDH-2 protein are absent. For complementation studies, the promoter region of the operon up to downstream of the stop codon of *gbs1788* was amplified by PCR with oligonucleotide pair 1788compFor and 1788compRev and chromosomal DNA of a *gbs1789* mutant (deletion mutant) and then cloned into EcoRI/BamHI sites of plasmid pTCV-lac (64). The resulting plasmid, pTCV-*ndh*^*C*^, was established in E. coli strain TG1 for sequencing and then in the Δ*ndh* mutant. pTCV-lac was used as a control. The plasmids were maintained by addition of kanamycin (Km) at 500 µg ml^−1^.

**TABLE 3:** Primers used in this study

Primer designation	Sequence (5′→3′)
gbs1788F	GATCGAATTCGTATTTTCTGGCTTGACAATGGG
gbs1788intR	GCTAGAAGTTCTTTAAGTCCACCGGCATAACCAGCACCTAAAACTAGG
gbs1788intF	CCTAGTTTTAGGTGCTGGTTATGCCGGTGGACTTAAAGAACTTCTAGC
gbs1788R	GATCGGATCCGTAAACATCCATAAACCAAGG
gbs1788extF	CTGCCTCTTTTATGGATGGG
gbs1788extR	GAGCCAAAAACCACTAGC
1788compFor	GCCGGAATTCCGGCTATTTAAAACAAATTGGAGC
1788compRev	CGGCGGATCCCGAAGAGCTAGTTTCCTAGCTTCG
FwNdhSNdeIHis	GGAATTCCATATGCATCACCATCACCATCACCATCACAAAGAAATCCTA GTTTTAGGTGC
RvNdhSHindIII	CCCAAGCTTTTAATGATATAAATCAAAACGTCCCTTAG

### Oxygen consumption assays and pH determination.

Oxygen consumption by S. agalactiae cells was determined at 37°C using a dual-channel respirometer system (model S782; Strathkelvin Instruments). The concentration of oxygen in the air-saturated buffer at 37°C was assumed to be 237.5 µM. Early-stationary-phase cells (8 h) were washed and resuspended in phosphate-buffered saline (PBS) buffer to an OD of ≈0.3 in a 1-ml chamber; oxygen consumption was monitored after addition of 0.2% glucose. Unless specified otherwise, the effect of different compounds on respiration was determined after 3-min incubations of the cells with the drugs at 37°C before addition of glucose.

The pH of the medium at 8 h of culture was determined using pH strips (MColorphast; Millipore).

### Determination of the NADH/NAD^+^ ratio.

Cells grown under respiration permissive or nonpermissive conditions were collected at early stationary phase (~8 h) by centrifugation at 14,000 × *g* for 5 min. Pellets were washed once with PBS and resuspended in 400 µl of extraction buffer at an OD of ~30. Samples were homogenized twice in a FastPrep-24 Beadbeater at 6 m/s for 45-s cycles with 2 min of incubation on ice in between. Homogenized samples were centrifuged at 4°C in a microcentrifuge at 14,000 × *g* for 2 min, and intracellular levels of NADH and NAD^+^ were measured using the NAD/NADH quantitation kit (MAK037; Sigma-Aldrich, St. Louis, MO) according to the manufacturer’s instructions. NADH and NAD levels were normalized to milligrams of protein.

### Hemolysis assay.

Hemolytic activity using whole bacteria was assayed as described by Nizet et al. (65). Briefly, cells grown until the early stationary phase (~8 h), in the presence or absence of 0.1% Tween 80 under respiration permissive or nonpermissive conditions, were pelleted by centrifugation at 14,000 × *g*, washed once with PBS, and resuspended in 1 ml of PBS with 0.2% glucose to an OD of ~1. In a 96-well conical-bottom microtiter plate, 100 µl of cell suspensions, in triplicate, was incubated with an equal volume of 1% sheep red blood cells (RBCs) in PBS-glucose. The plate was incubated at 37°C for 1 h and then spun down at 640 × *g* for 5 min to pellet unlysed RBCs and bacteria. The supernatants were transferred to a replica 96-well plate, and hemoglobin release was measured by recording the absorbance at 420 nm. RBCs in PBS-glucose and RBC lysis with 0.1% sodium dodecyl sulfate (SDS) were used as negative and positive controls, respectively.

### Animal assays.

Six-week-old female BALB/c mice were purchased from the Animal Resources Centre (Australia). A systemic infection model was used to assess virulence of S. agalactiae. Bacteria used for infection were grown statically in Todd-Hewitt broth (THB) overnight at 37°C. Mice were inoculated through the lateral tail vein with 2 × 10^6^ bacteria in PBS (pH 7.4); inocula were delivered in 200 µl using 27G × 1^1^/_4_-in. PrecisionGlide needles (BD) connected to 1-ml tuberculin syringes. At various intervals, groups of five mice were sacrificed, and blood was collected by cardiac puncture. Organs were collected, weighed, and homogenized in PBS. The bacterial numbers in blood and organ homogenates were determined by plating on THB agar plates incubated at 37°C. Statistical analysis of bacterial counts was performed using the Kruskal-Wallis test, followed by Dunn’s multiple comparisons. GraphPad Prism (version 7.0b) software was used for all statistical analyses. A *P* value of <0.05 was considered statistically significant. The animal experiments were repeated twice in independent experiments and were approved by Griffith University Animal Ethics Committee (approval no. MSC/01/15/AEC).

### NDH-2 heterologous expression and purification.

The S. agalactiae NDH-2 (*ndh* [*gbs1788*]) open reading frame was amplified from NEM316 using primers listed in Table 3 and cloned into the pET22-b expression plasmid, with an N-terminal 8×His tag. The E. coli c43(DE3) strain, carrying the pRARE plasmid and transformed with the resulting plasmid, was grown under aerobic conditions (200 rpm) at 37°C in LB medium supplemented with 50 µg/ml of kanamycin and 100 µg/ml of ampicillin until an OD_600_ of ≈0.8, and expression was induced with 1 mM IPTG (isopropyl-β-d-thiogalactopyranoside) for 4 h. All subsequent steps were performed at 0 to 4°C. Cells were harvested at 14,000 × *g* for 10 min and resuspended in buffer A (50 mM sodium phosphate buffer [pH 7.5], 300 mM NaCl) plus 5 mM MgSO_4_, DNase I, and protease inhibitor cocktail (Sigma). These cells were then disrupted by passing three times through a microfluidizer at a pressure of 80,000 lb/in^2^. The resulting extracts were centrifuged at 14,000 × *g* for 10 min to remove unbroken cells, and supernatants were subject to ultracentrifugation at 230,000 × *g* for 4 h to obtain membrane pellets. Membrane fractions were resuspended in buffer A plus the protease inhibitor cocktail and solubilized by addition of a stock solution of 20% dodecyl-β-d-maltoside (DDM) dropwise to a final concentration of 1%. The suspension was incubated at 4°C for 1 h with mild agitation and then cleared by centrifugation at 230,000 × *g* for 1 h. Solubilized membranes were added to 5 ml of Ni-nitrilotriacetic acid (NTA) resin (Qiagen Sciences, Germantown, MD) preequilibrated with buffer A plus 10 mM imidazole and 0.05% DDM. The protein bound to the resin was washed with buffer A plus increasing concentrations of imidazole (10 to 50 mM) and 0.05% DDM. Protein was eluted with buffer A plus 200 mM imidazole and 0.05% DDM and concentrated by filtration (Millipore concentrator). Imidazole was removed by a series of filtration and washing steps with buffer A plus 0.05% DDM. The purified protein was stored frozen at −80°C after addition of glycerol to a final concentration of 10%.

### Biochemical characterization.

Protein concentration was determined using the Pierce bicinchoninic acid (BCA) protein assay kit. Purity of the sample was observed by running SDS-PAGE. The FAD concentration was measured by addition of 5% trichloroacetic acid followed by centrifugation and determination of supernatant absorbance at 450 nm (ε_450_: 11,300 mM^−1^ cm^−1^) (29).

### Enzyme activity assay and determination of kinetic parameters.

The rate of NADH oxidation was determined using a UV-Vis spectrophotometer (Agilent Technologies model 8453) by monitoring absorbance at 340 nm (NADH oxidation) upon addition of 100 µM NADH in the presence of 2 nM enzyme and a 100 µM concentration of a quinone soluble analogue. Assays were performed at 37°C in buffer B (50 mM sodium phosphate buffer [pH 7], 150 mM NaCl) plus 125 µg ml^−1^ of soy asolectin (Sigma) and 20 µM FAD. For determination of kinetic constants, 2 to 200 µM NADH and 2 to 200 µM of DMN were added to the reaction mixture containing 2 nM enzyme. Enzyme rates are expressed as a turnover number (*k*_cat_) based on moles of NADH oxidized per second per mole of enzyme.

### Inhibitor screening.

Isolated NDH-2 activity was tested against 1,905 compounds from two FDA-approved drug libraries (Selleck Chemicals, with 1,176 compounds, and the NIH Clinical Collection [NCC], with 729 compounds). Enzyme activity was measured in buffer B with 125 µg ml^−1^ of soy asolectin, 20 µM FAD, and 50 µM MD, in the presence and absence of 25 µM each compound. The reaction was started upon addition of 100 µM NADH, after a 3-min incubation of the reaction mixture at 37°C. The compounds found to inhibit at least 80% of enzyme activity *in vitro* were further selected to determine their 50% inhibitory concentration (IC_50_) and GI_50_. IC_50_ refers to the concentration that causes 50% inhibition of enzyme activity, while GI_50_ represents the concentration that causes 50% inhibition of cell growth. The IC_50_ for each compound was determined by testing enzyme activity after a 3-min incubation in the presence of different concentrations of the inhibitor, before starting the reaction by addition of NADH. GI_50_ was determined by growing the bacterial cells overnight in the presence of different concentrations of each inhibitor under respiration permissive growth conditions.
