# Supplementary figures and images for: Type 2 NADH Dehydrogenase Is the Only Point of Entry for Electrons into the Streptococcus agalactiae Respiratory Chain and Is a Potential Drug Target
Source: mBio. 2018 Jul 3;9(4):e01034-18. doi: 10.1128/mBio.01034-18 (PMC6030563; doi:10.1128/mBio.01034-18)

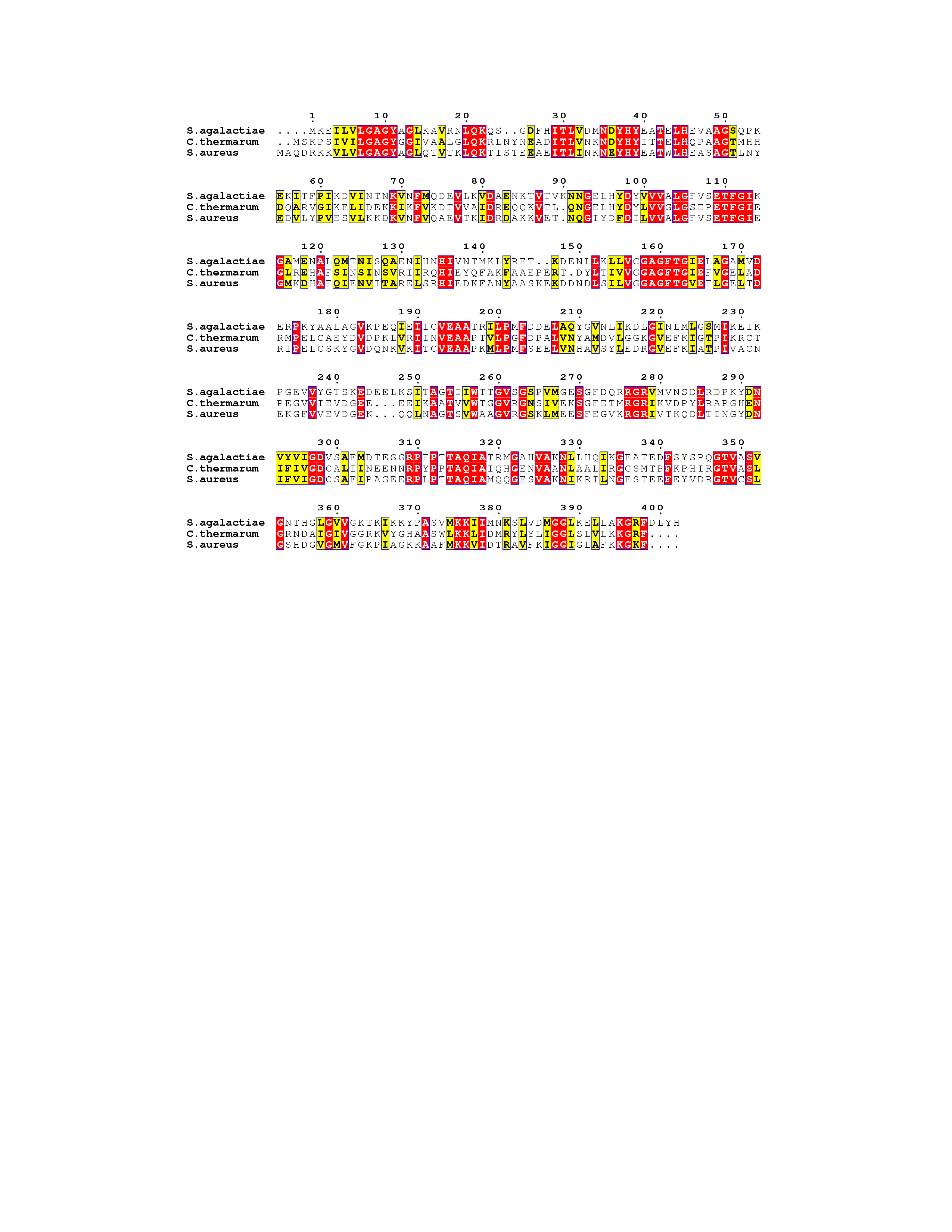

Supplement: FIG S1 [file mbo004183959sf1.tif]

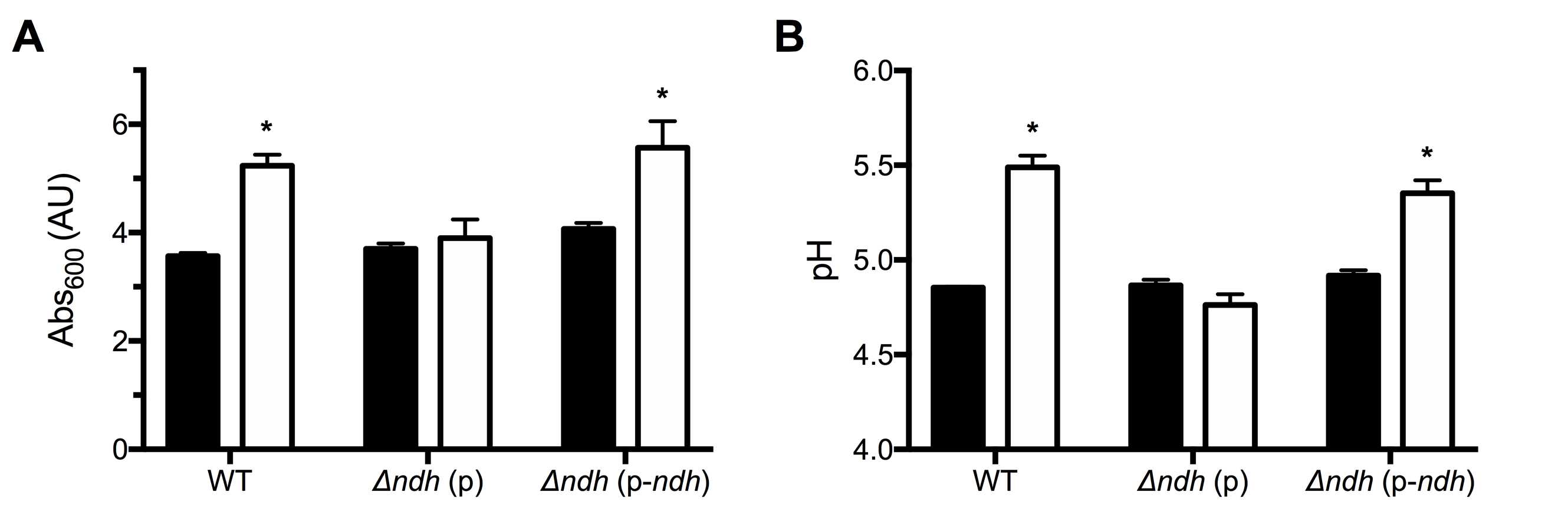

Supplement: FIG S2 [file mbo004183959sf2.tif]

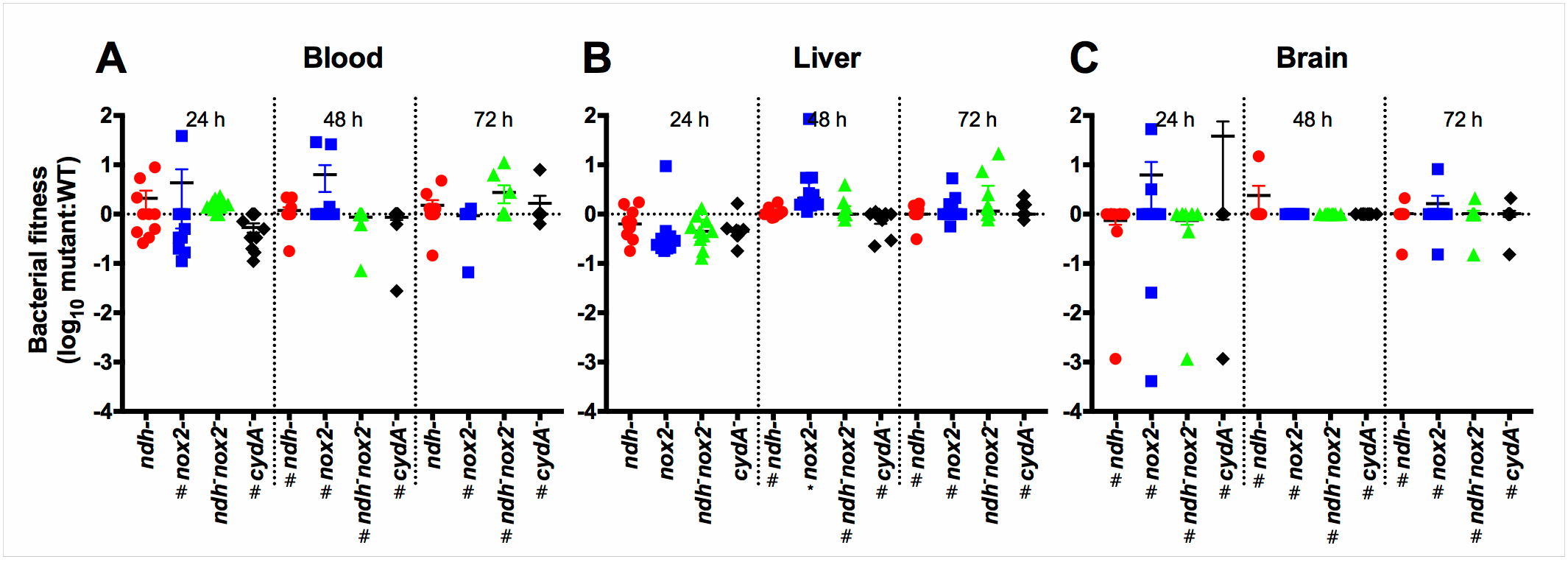

Supplement: FIG S3 [file mbo004183959sf3.tif]
